# Molecular Markers in Urinary Bladder Cancer: Applications for Diagnosis, Prognosis and Therapy

**DOI:** 10.3390/vetsci9030107

**Published:** 2022-02-28

**Authors:** Ana Mafalda Rasteiro, Eva Sá e Lemos, Paula A. Oliveira, Rui M. Gil da Costa

**Affiliations:** 1 CEDIVET, Laboratório Clínico Veterinário, 4200-071 Porto, Portugal; ana.rasteiro@gardenvets.co.uk (A.M.R.); evasalemos@gmail.com (E.S.e.L.); 2 Garden Veterinary Group, Chippenham SN15 1NQ, UK; 3 Centre for the Research and Technology of Agro-Environmental and Biological Sciences (CITAB), Inov4Agro, University of Trás-os-Montes e Alto Douro (UTAD), Quinta de Prados, 5000-801 Vila Real, Portugal; pamo@utad.pt; 4 Molecular Oncology and Viral Pathology Group, Research Center of IPO Porto (CI-IPOP)/RISE@CI-IPOP (Health Research Network), Portuguese Oncology Institute of Porto (IPO Porto)/Porto Comprehensive Cancer Center (Porto. CCC), 4200-072 Porto, Portugal; 5 Postgraduate Programme in Adult Health (PPGSAD), Department of Morphology, University Hospital (HUUFMA), Federal University of Maranhão (UFMA), São Luís 65080-805, Brazil; 6 LEPABE—Laboratory for Process Engineering, Environment, Biotechnology and Energy, Faculty of Engineering, University of Porto, Rua Dr. Roberto Frias, 4200-465 Porto, Portugal

**Keywords:** transitional cell carcinoma, urothelial carcinoma, histology, therapeutic marker, prognosis

## Abstract

Cancer of the urinary bladder is a neoplasm with considerable importance in veterinary medicine, given its high incidence in several domestic animal species and its life-threatening character. Bladder cancer in companion animals shows a complex and still poorly understood biopathology, and this lack of knowledge has limited therapeutic progress over the years. Even so, important advances concerning the identification of tumour markers with clinical applications at the diagnosis, prognosis and therapeutic levels have recently been made, for example, the identification of pathological *BRAF* mutations. Those advances are now facilitating the introduction of targeted therapies. The present review will address such advances, focusing on small animal oncology and providing the reader with an update on this field. When appropriate, comparisons will be drawn with bladder cancer in human patients, as well as with experimental models of the disease.

## 1. Prevalence and Aetiology

Urinary bladder cancer represents about 2 percent of all reported canine malign neoplasms [1,2], and it is rare in cats [3,4]. The majority of canine bladder tumours are malignant and of epithelial origin [5]. Transitional cell carcinoma (TCC), also referred to as urothelial carcinoma (UC) is the most frequent canine urinary bladder tumour [6]. The aetiology of the canine disease is thought to be multifactorial. Several risk factors have been proposed to play a role, such as exposure to older topical insecticides for flea and tick control, obesity, female sex, herbicides and breed predisposition (e.g., Scottish Terrier, West Highland White Terrier, Shetland Sheepdog, Beagle and others) [7,8,9].

Urinary bladder tumours are also frequently observed in cattle grazing on pastures infested by toxic ferns (mainly *Pteridium* spp.) (reviewed by Gil da Costa et al., 2012 [10]) and have been reported in other ruminant species [11]. The aetiology of bladder cancer in ruminants is much clearer than in companion animals. Grazing on poisonous ferns has been identified as a decisive risk factor since the mid-1900s [12,13]. The occurrence of bladder tumours in cattle is closely related to the geographical distribution of toxic ferns and originates in a syndrome known as bovine enzootic haematuria. Bladder lesions have also been reproduced experimentally in multiple laboratory animal models, by administering the fern or its toxin ptaquiloside [14,15,16,17,18]. Ptaquiloside is a DNA-alkylating agent, which causes point mutations, as well as structural and numeric chromosomal aberrations [19,20,21]. This toxin also has immunotoxic properties, contributing to reducing immune surveillance against newly arising neoplasms [22,23,24]. Other ferns containing ptaquiloside (e.g., *Pteris* spp. and *Dryopteris* spp.) or structurally related illudane toxins (for a review of illudane toxins, see Gil da Costa et al., 2013 [25]) do occur and have been reported to cause bladder cancer in cattle in various locations worldwide [26,27]. Bracken consumption has been proposed to facilitate a persistent abortive infection of the bovine bladder by bovine papillomavirus (BPV) types 1, 2, 13 and 14 [28,29,30,31]. These Delta BPV types are hypothesised to contribute to bladder carcinogenesis, by activating the platelet-derived growth factor receptor beta (PDGFR-β) through their oncoprotein E5 [32,33]. 

## 2. Histology and Grading

The urothelium is a hierarchically organised tissue, comprising basal, intermediate and umbrella cells, and the development of urothelial cancers progressively subverts this normal hierarchical structure [34]. Data obtained from human patients and from laboratory animals have helped trace different types of urothelial carcinoma to specific cell populations of origin and different differentiation pathways [35,36,37,38]. Although the pathogenesis of canine bladder cancer is less clear, it seems that numerous genetic changes involving key genes are shared between human and dogs, reflecting a conserved mechanism of pathogenesis [39]. Current laboratory models of bladder cancer, based on rats and mice, are out of the scope of the present work, but several comprehensive reviews have been recently published [40,41].

About 90 percent of all urinary bladder tumours in dogs are epithelial and malignant, and 50 to 90 percent of these will metastasise. Urinary bladder carcinomas include transitional cell carcinoma, squamous cell carcinoma, adenocarcinoma and undifferentiated carcinoma (Table 1) [2]. Among primary epithelial neoplasms of the urinary bladder, TCC represents 75 to 90 percent in dogs [2]. More than 50 percent of overall malignant tumours show involvement of both the bladder and the urethra [5]. Benign epithelial tumours are rare, and only 10 percent of canine urinary bladder tumours are of mesenchymal origin, with smooth muscle neoplasms being the most common [2].

Canine transitional cell carcinomas are classified based on their growth patterns (Table 2). They can be divided into papillary (papillary or cauliflower exophytic growths projected into the lumen) and non-papillary (plaques, flat nodules or masses), and into infiltrating or non-infiltrating tumours [2]. The consistent observation is that the majority (90 percent) of canine TCC shows an infiltrating growth pattern [2]. Papillary infiltrating TCC is one of the most common variants and likely to metastasise. The non-papillary and infiltrating type is the second or the most common variant in dogs, depending on the study, and shows a high tendency to metastasise [2].

In cattle, the histological features of bladder tumours are quite different, with 51.2% of purely epithelial tumours, 17.4% of purely mesenchymal tumours and 31.4% of coexisting epithelial and mesenchymal tumours, and numerous benign tumours (papillomas, haemangiomas, etc.) [42].

Over the years, several different grading systems for urothelial carcinomas in humans have been proposed and applied to veterinary tumours, looking for a better approach on the evaluation of the tumours’ biologic behaviour [2,43]. Meuten and Meuten (2016) proposed a more simplified classification of TCC into low or high grade (Figure S1, Supplementary Material). The majority of canine TCC are invasive, high grade and at an advanced stage when diagnosed [2]. In affected dogs, high-grade tumours seem to be more common in terriers than in non-terrier breeds [44].

## 3. Diagnosis

### 3.1. Clinical Signs and Differential Diagnosis

Clinical signs in dogs with TCC are usually nonspecific, many of which, such as dysuria, haematuria and pollakiuria, are commonly observed with urinary tract disease [2,5]. Concurrent urinary tract infections (UTI) are often present [2]. Tumour growth can lead to obstruction of the ureters or urethra and invasion and disruption of the normal functioning of the urethral sphincter [45]. On physical examination, a thickening of the urethra and of the trigone region of the bladder and enlargement of iliac lymph nodes may be found and, occasionally, a mass in the bladder or a distended bladder [1]. Urinary tract obstruction can occur prior to the development of lethal metastasis and is a common cause of death in dogs with TCC [6]. However, a normal physical examination does not exclude the presence of a TCC [46]. Differential diagnoses of canine TCC comprise other neoplasia, chronic cystitis, polypoid cystitis, fibroepithelial polyps, granulomatous cystitis/urethritis, calculi, among others [1].

In cattle, haematuria and weight loss are the main symptoms of bladder cancer and often present as part of the previously mentioned syndrome, known as bovine enzootic haematuria [26,47].

### 3.2. Diagnostic Procedures and Staging

Diagnostic procedures for TCC should include a complete blood cell count, serum biochemistry profile, urinalysis, urine culture (to rule out lower urinary tract infection) and cancer staging [1,46]. 

Definitive diagnosis of TCC can be established via histopathologic examination of tumour tissue and/or cytology of a representative sample [2]. Biopsies can be collected by means of cystotomy, cystoscopy and traumatic catheterisation, invasive procedures that usually involve general anaesthesia [1,39]. Cytological samples may be obtained by direct or ultrasound-guided percutaneous mass fine-needle aspirate or traumatic catheterisation [48]. 

The risk of tumour implantation or seeding/dissemination throughout other tissues following diagnostic or therapeutic procedures has been reported, especially after surgical manipulation of the tumour [49,50,51,52]. Even though reports are scarce, these should be carefully interpreted. Where possible, less invasive techniques should be favoured.

A less invasive technique consists of performing a cytology from urine sediment. If tumour cells are present, a diagnosis can be achieved [2]. However, negative results do not rule out TCC. In one study, malignant cells were seen in only 30% of dogs with lower urinary tract tumours [5]. Thus, cytological results must be interpreted with caution, especially upon the presence of inflammation of the urinary tract, and correlation with clinical data is essential for reaching a diagnosis.

Clinical staging of canine TCC includes thoracic and abdominal radiography, abdominal ultrasonography and specific urinary tract imaging [1,46]. Computer tomography (CT) has increasingly been used to aid in diagnostics and staging, particularly for more accurately evaluating the urethra and to detect metastases [53]. Figure S2 (Supplementary Material) shows the TNM (tumour, node, metastasis) classification for clinical staging of canine bladder cancer [54]. The TNM stage at diagnosis for TCC has shown to be strongly related to prognosis. More advanced TNM stage at the time of diagnosis was significantly associated with shorter survival [7,53]. Tumours located in the urethra were also associated with shorter survival time than ones in the bladder [53]. TCC has rarely been curable; however, with current therapies, many dogs will achieve stable disease for several months after diagnosis [1].

### 3.3. Recent Advances in Diagnostic Techniques for UC

As mentioned above, clinical presentation of canine TCC is comparable to several other (and far more common) urinary tract disorders. Consequently, the diagnosis of TCC is often delayed, allowing the tumour to grow, infiltrate and metastasise [2]. In fact, most TCCs are currently not diagnosed until they reach an advanced stage and thus present poor prognosis [55]. As such, effective (and preferably less invasive) methods for the early identification of UC are needed, which could improve responses to treatment and survival rates among affected dogs [56]. In particular, because TCC tumour cells and metabolites may be shed into urine, this body fluid is likely to present tumour-specific molecules that could be used as biomarkers for tumour detection using easily accessible samples collected through non-invasive techniques [46,57]. Over recent years, several potential biomarkers for canine TCC have been investigated for diagnostic/screening or prognostic purposes, and a few of them are currently available for commercial use. 

These and other potential biomarkers are summarised in Table 3 and detailed below.

#### 3.3.1. BRAF Mutation

The somatic *BRAF^V595^^E^* mutation in canine *Canis familiaris* chromosome 16 (CFA16) results in a valine to glutamic acid substitution at codon 595 of canine *BRAF* [58,59]. 

In one study, the *BRAF^V595E^* mutation was detected across 87.9% of canine invasive TCC bladder tumour tissue samples (58 out of 66 samples). The mutation was also detected in the urine sediments of dogs tested with mutation-positive tumours (9/9), and none of the healthy control dogs were positive for the mutation [58]. Another study screened for the presence of the *BRAF* mutation in 667 canine tumours, including a series of haematopoietic tumours (*n* = 245), sarcomas (*n* = 160), carcinomas (*n* = 115), melanocytic tumours (*n* = 72), as well as other, less common cancers (*n* = 75). The *BRAF* mutation was identified in 64 primary tumours (9.6%) of different origins with various frequencies, with particularly high frequency in prostatic carcinoma (PC) (20/25, 80%) and urothelial carcinoma (30/45, 67%) tissue samples. No mutation was found in normal control tissues [59].

Furthermore, a droplet digital PCR (ddPCR) assay for detection of the canine *BRAF^V595E^* mutation in canine urogenital tumours was developed. Tested samples included tissue obtained from TCC, PC and non-neoplastic bladder epithelium, and free-catch urine samples derived from dogs with UC, PC, with cystitis and healthy controls. In tissue samples, 75% of TCC (36/48), 85% of PC (23/27) and none of control samples were mutation positive. The mutation was also identified in urine samples from 83% (19/23) of canine TCC and 100% of PC patients (3/3), and none of control samples were positive for the mutation. The V595E mutation was consistently detected in matched tissue and urine specimens from six of these dogs [60]. 

Grassinger and their team (2019) studied the presence of *BRAF* mutation in TCC tissue samples from 65 dogs from different breeds (both terriers and non-terriers). Overall, the mutation was detected in over half of the specimens, with considerably higher prevalence in terriers compared to the other breeds (73% versus 36%) [44]. Parker et al. (2020) confirm this hypothesis of hereditary predisposition to TCC [61]. Histological grade, however, was not significantly correlated with the *BRAF* mutation [44]. 

A recent retrospective study looked at the prognostic significance of *BRAF* mutation in 79 canine TCCs. A total of 51 tumours (65%) were *BRAF^V595E^*-positive, and the mutation was not correlated with survival [62]. 

Cells, both normal and tumoural, can release DNA into the bloodstream (circulating cell-free DNAs) via processes such as direct secretion, necrosis and apoptosis. Circulating tumour DNAs (ctDNA) contain the same genetic information as the original tumour, and they have been investigated in human and veterinary medicine for various applications, including studying tumour features and monitoring disease progression and response to treatment [106,107,108,109,110].

In a study including 15 dogs with TCC, 11 (73%) were positive for *BRAF* mutation, which was assessed in either tissue or urine samples. Blood samples were collected, and cfDNA was obtained from plasma. Results showed that concentrations of *BRAF*-mutated ctDNA were higher in *BRAF^V595E^*-positive dogs than in wild-type dogs. However, they did not correlate with clinical stage or the presence of metastases. Furthermore, six of the dogs were monitored throughout treatment and course of the disease. Levels of mutated *BRAF* ctDNA increased with disease progression and decreased with response to treatment. It is noteworthy that *BRAF* mutation could be present in other neoplasms and therefore contribute to the overall levels of ctDNA detected. Additionally, treatment therapies varied between analysed patients [63]. Further validation studies are encouraged.

Overall, these findings suggest that detection and quantification of the *BRAF* mutation in canine TCC patients, especially through non-invasive techniques, could be very useful in the clinical setting, not only as a biomarker for diagnosis, but also for monitoring disease progression and treatment response. A test, which based on ddPCR, is currently commercially available for use in urine samples and could be particularly useful as a screening test (Table 3) [60,64]. 

#### 3.3.2. Bladder Tumour-Associated Antigen Test (BTA)

The BTA was originally developed for use in humans (Bard BTA test). It was then assessed in canine patients, and a veterinary version (V-BTA) of the test was also developed. It consists of a rapid latex agglutination dipstick colorimetric test that allows the qualitative detection of tumour analytes in urine. The test uses antibodies to detect a urinary bladder tumour-associated glycoprotein complex. This complex contains basement membrane proteins that are degraded upon urinary tract invasion or tissue damage and shed into the urine and may also contain immunoglobulins [68,69]. Tests results are either positive or negative.

Studies performed with canine cohorts evaluated: 65 animals, including patients with TCC (associated with the bladder, urethra, prostate or vagina), as well as healthy controls and urologic controls (e.g., dogs with other urologic conditions or with systemic disease leading to abnormal urinalysis examination) [70]; 54 animals, including dogs with lower urinary tract neoplasia (TCC or other neoplasia affecting the bladder and/or other urogenital structures), dogs without urinary tract abnormalities and dogs with non-malignant urinary tract disease [71]; 229 animals, including dogs with TCC of the lower urinary tract (involving the urinary bladder and/or other structures), healthy control dogs, unhealthy control dogs with (non-TCC) urinary tract disease and unhealthy control dogs without urinary tract disease [68]; 73 animals, including dogs with TCC (of the bladder, prostate and/or urethra, with or without concomitant UTI), healthy dogs, proteinuric dogs and dogs with non-TCC associated lower urinary tract disease (LUTD) [72]. Overall, the test exhibited high sensitivity but lower specificity for the detection of TCC in dogs. Therefore, the V-BTA has not been recommended as a confirmatory/definitive diagnostic test for urinary tract TCC in dogs and should not be indiscriminately used in every patient presenting clinical signs of urinary tract disease. However, it can be useful as a screening test to rule out TCC, especially in dogs at high risk of developing the disease. As an example, considering the prevalence of TCC in a population of geriatric dogs, less than 3% of dogs with positive V-BTA test results would be expected to have TCC. On the other hand, 99.9% of dogs with a negative test would not have the disease [68]. 

#### 3.3.3. Basic Fibroblast Growth Factor (bFGF)

The basic fibroblast growth factor (bFGF) is a proangiogenic peptide that has been associated with tumour progression in humans and has been detected at high levels in the urine of humans with urologic and non-urologic malignancies [73]. An ELISA test kit for human bFGF that recognises natural and recombinant bFGF has been developed and has also been used for the quantification of canine bFGF in urine [74,75,76]. 

In one study, bFGF levels were quantified in the urine of dogs with locally active TCC of the urinary bladder (*n* = 7) and compared with those in dogs with urinary tract infection (*n* = 10) and normal dogs (*n* = 17). bFGF concentrations were significantly higher in dogs with bladder cancer than in normal dogs and in dogs with UTI, while these two groups showed comparable results. The study suggested that urine bFGF could be useful as a diagnostic tumour marker, allowing for distinction between dogs with UTI and dogs with TCC, or as a non-invasive indicator of treatment response [73]. In another study, urinary bFGF concentrations were measured in 14 dogs with invasive TCC of the urinary bladder (with or without involvement of the urethra or the prostate) before and after treatment with piroxicam. Before treatment, urine bFGF levels were significantly higher than those of eight normal dogs. In 77% of dogs (11 out of 14), bFGF concentrations decreased with treatment, and tumour volume decreased by 9–75% in 10 of those 11 dogs. Overall, a positive correlation was seen between the change in urine bFGF concentration and the change in tumour size [77]. In a similar study, bFGF concentrations were assessed in the urine of dogs with bladder TCC before and after treatment with piroxicam/cisplatin. Before treatment, concentrations of bFGF were significantly higher than those in normal dogs (eight animals per group). Concentrations decreased with treatment in four out of eight dogs, and tumour volume decreased by 59–95% in these four dogs. On the other hand, concentrations increased in four dogs, two of them also showing an increase in tumour size. No significant association was observed between change in urine bFGF concentration and change in tumour size [78]. Additional studies are required for determining the sensitivity and specificity of this test in a larger canine population and its potential use to determine disease progression, clinical stage and response to treatment. 

#### 3.3.4. Chromosomal Copy Number Aberrations (CNAs)

Fluorescence in situ hybridisation (FISH) is considered the gold standard of copy number detection and enumeration. In biopsies from canine TCC, chromosomal copy number aberrations (CNAs) have been identified throughout several regions of the canine genome. In fact, three of those CNAs are highly recurrent features: *Canis familiaris* (CFA) chromosome 13 gain, CFA 36 gain and CFA 19 loss. 

FISH was shown to be effective in identifying aberrant cells in urine sediment samples from dogs with TCC confirmed via urine cytology or histopathology. In a study involving 24 dogs diagnosed with UC, all animals showed at least one of these three aberrations in cells recovered from urine sediment specimens [39]. Thus, the detection of these aberrations may potentially be used as a molecular diagnostic test for TCC. In the future, validation of the technique with a larger canine population is needed, including dogs presenting with non-neoplastic urinary diseases, in order to establish true sensitivity and specificity values for a clinically valuable diagnostic assay [39]. An in vitro test is available for use in human samples [79].

A multiplexed droplet digital polymerase chain reaction (ddPCR) assay has also been developed for detection and quantification of copy number imbalances/changes characteristic of canine TCC. Aimed to detect CNAs of specific regions of canine chromosomes 13, 19 and 36, the assay proved effective at differentiating 31 neoplastic and 25 non-neoplastic bladder tissues (including normal bladder and bladder with non-neoplastic lesions), based on copy number, with 100% sensitivity and specificity. When evaluated in DNA isolated from free-catch urine samples from TCC and non-TCC dogs, copy number imbalance (for CFA 13 and CFA 36) was detected in 67% (12 out of 18) TCC specimens and was absent in non-TCC urine samples (*n* = 7, including clinically healthy dogs and those with urinary tract infections). This assay was shown to be a fast method with a potential clinical value for determination of copy imbalance not only in tissue samples but also in free-catch urine from dogs diagnosed with UC. Further evaluation of the ddPCR assay as a molecular diagnostic test for canine TCC in a clinical setting is required, with a larger sample including both TCC and non-neoplastic urinary diseases, in order to assess its sensitivity and specificity [55]. A test for use in urine clinical samples has recently been developed (Table 3) [64].

#### 3.3.5. Microsatellite Instability

Microsatellites (MS) are short tandem repeats of DNA that occur mainly in noncoding regions [111]. Microsatellite instability (MSI) refers to the accumulation of mutations within MS, and it can promote tumourigenesis. A panel of 22 MS sequences was evaluated by PCR amplification of DNA samples extracted from exfoliated urothelial cells and blood cells. Samples included urine from dogs with TCC (of the bladder, prostate and urethra, diagnosed by fine-needle aspiration cytology or histologic evaluation) and from control dogs (proteinuric dogs, dogs with lower urinary tract disease not associated with TCC and healthy controls). The study showed that detection of these MS abnormalities could be achieved in canine urine samples. MSI was more frequently detected in urine samples from dogs with TCC (55%, *n* = 11 out of 20) compared with control dogs (32%, *n*= 12 out of 38). However, the difference was not statistically significant. Measurement of MSI in urine was poorly specific and not sensitive for identification of TCC in dogs [72]. 

Further research is needed to determine whether targeting other specific loci might improve the diagnostic utility of the test and to determine whether MSI represents an early preneoplastic change and can be an indicator of urothelial carcinogenesis in canine TCC [72].

#### 3.3.6. MicroRNAs (miRNAs)

MiRNAs are small endogenous noncoding RNAs involved in post-transcriptional regulation of gene expression that take part in most biological processes in mammals, such as cell proliferation, differentiation and apoptosis. Dysregulation of miRNAs plays a significant role in cancer development [112,113]. 

A study measured the expression of specific miRNAs that have the ability to target components of the p53, Rb and Bcl-2 pathways, which are involved in the development of bladder tumours in humans. Five miRNAs (miR-34a, let-7c, miR-16, miR-103b and miR-106b) were quantified in bladder tissue samples from dogs with grossly normal urinary bladders, non-neoplastic inflammatory bladder disease and with TCC, as well as in five established TCC cell lines. Expression of miR-16, miR-34a, miR-103b and miR-106b was up-regulated in TCC samples, with significantly higher expression compared with bladder samples obtained from dogs with inflammatory lower urinary tract disease. The levels of miR-34a and miR-106b in TCC samples were also significantly increased compared to those in grossly normal bladder. The study concluded that miR-34a, miR-16, miR-103b and miR-106b could potentially be oncogenic in TCC and could be useful diagnostic biomarkers for the identification of dogs with the disease. One limitation of the study was the fact that only samples from muscle-invasive TCC were analysed, with many of the patients being at an advanced or metastatic stage [56].

In another study, expression levels of the five miRNAs were also evaluated by qPCR, in blood and urine (sediment) samples from 70 dogs with clinically normal bladders (*n* = 28), inflammatory or infectious lower urinary tract diseases (LUTD, *n* = 25) and TCC (*n* = 17). In blood samples, statistically significant differences were found in the expression levels of miR-103b in normal patients compared to LUTD and with TCC, but no significant differences were identified between LUTD and TCC patients in any of the five miRNAs levels. Conversely, in urine samples, significant differences were found in miR-103b and miR-16 levels between LUTD and TCC dogs. Additionally, differences were detected in urine levels of let-7c in normal versus LUTD and TCC patients, and in miR-103b and miR-106b in normal versus TCC patients. Data also revealed alterations in the coordinated expression (expression trends) of miRNAs in urine from patients with LUTD and TCC. Both miR-103b and miR-16 were suggested as potential non-invasive diagnostic biomarkers for TCC, particularly for distinguishing LUTD and TCC in canine urine samples [80]. However, additional research is required to evaluate diagnostic sensitivity of the method, to determine the potential role and changes in expression of miRNAs during development and progression of TCC, and to investigate their potential as therapeutic targets.

#### 3.3.7. Telomerase

Telomeres are repeated DNA sequences located at the end of chromosomes, which, with each cellular division, are partly lost and become shorter, a process that is related to ageing and cell death. Telomerase reverse transcriptase is frequently up-regulated in cancer cells and is responsible for the preservation of telomere ends, an important mechanism by which tumour cells escape senescence [114,115]. 

The activity of this enzyme was measured in canine samples by using an in vitro PCR-based telomeric repeat amplification protocol (TRAP) developed by Kim et al., (1994) [81,82]. Telomerase activity was detected in a canine TCC cell line, as well as in urine samples collected from 10 out of 11 dogs with TCC and from 2 out of 10 dogs with benign lower urinary tract disease. Activity of this enzyme was not detected in any of the healthy dogs. Samples were obtained from dogs with TCC (located in the bladder or the prostate; confirmed by histology/cytology of the mass; some dogs were receiving treatment for TCC at the time of sample collection), dogs with benign lower urinary tract disease and healthy dogs (*n* = 30). The technique was shown to be highly sensitive in detecting telomerase activity. However, urine samples containing other telomerase-positive cells could yield false-positive results (e.g., presence of activated lymphocytes in dogs with bacterial cystitis) [82]. 

The study concluded that telomerase activity may be a useful diagnostic marker for canine urothelial carcinomas, and specifically, the TRAP assay may be useful in diagnosing canine TCC in clinical urine samples/in a clinical context. Additional studies are required to more accurately determine the performance of the test in larger populations, including more dogs with varying degrees of benign LUTDs, to further establish the specificity of the test. Further research is also needed on optimisation of urine storage and processing protocols in order to develop an accurate non-invasive diagnostic test for evaluation of clinical samples [82].

#### 3.3.8. Calgranulins

Calgranulins are innate antimicrobial proteins belonging to the S100 family of calcium-binding proteins that include the S100A8/A9 complex (also called calgranulin A/B or calprotectin) and S100A12 (calgranulin C). They are expressed by cells of the innate immune system and have been associated with inflammatory disorders. Calprotectin is also expressed by epithelial cells upon malignant transformation and plays a role in the regulation of cell proliferation and metastasis. This protein complex is overexpressed in human bladder and prostate cancers, whereas results for S100A12 have not been clear [2,83]. 

A method based on species-specific radioimmunoassays that was established and validated by Heilmann and co-authors [46] was conducted to measure urine concentrations of canine calgranulins S100A8/A9 and S100A12 [83]. Urine samples (*n* = 239) were collected from dogs with TCC/PC (treated and treatment-naïve), non-neoplastic urinary tract disease, other neoplasms, UTI and healthy controls. Tumour locations included urinary bladder, urethra and the prostate, whose diagnoses were confirmed by either cytology or histopathology. Results were presented as normalised to urine specific gravity levels (S100A8/A9USG) and as S100A8/A9-to-S100A12 ratio (UCalR). The study concluded that S100A8/A9USG and UCalR could be useful for diagnosing canine TCC/PC. S100A8/A9_USG_ could be a good a screening test for TCC/PC in dogs, especially in those where a UTI has been ruled out as a cause of clinical signs of lower urinary tract disease (due to a moderate rate of false positives observed for dogs ≥ 6 years of age with UTI). The uCalR can, in turn, help differentiate patients with a UTI from those with TCC/PC, even though a moderate false negative rate was seen in dogs ≥ 6 y.o. with a UTI. A combination of S100A8/A9_USG_ and uCalR improved diagnostic accuracy for the detection of canine TCC/PC. 

In this study, the possibility of occult urinary tract diseases, including TCC/PC, could not be completely excluded in the healthy control group. So, validation of these results in a larger cohort is encouraged. Further investigation is also required to explore a potential correlation between urinary expression/concentrations of the S100/calgranulins and tumour grade, staging, treatment response, progression and survival time [83].

#### 3.3.9. Proteomics

A preliminary study characterised the proteome of canine urine samples by using liquid chromatography tandem mass spectrometry (LC-MS/MS). Three cohorts were included, comprising four animals each: healthy dogs, dogs with UTI and dogs with TCC (located in the bladder, some of them with urethral and ureter involvement; confirmed via cytology or histology; treated or untreated for TCC). Of the 379 proteins identified in urine samples, 96 were present exclusively in the TCC group. The study identified a protein signature that could distinguish between healthy patients and those with TCC or UTIs. A statistical model using a biomarker multiplex for categorising samples as TCC or non-TCC was developed, predicting the presence of disease with 90% confidence. Further analyses of larger cohorts are required to confirm the relevance of the identified proteins as biomarkers for the diagnosis of TCC in dogs. Development of a more direct assay for the detection of the specific proteins will be useful for clinical diagnosis [84].

#### 3.3.10. Metabolomics

Metabolomics (also known as metabolic profiling) is an approach that involves the identification and global analysis of metabolite concentrations in cells, tissues or organisms [116]. Metabolite profiling analysis was performed on urine samples obtained from dogs with naturally occurring invasive TCC of the urinary bladder and/or urethra (*n* = 40, diagnosed by histopathology) and from healthy control dogs (*n* = 42). Proton nuclear magnetic resonance (NMR) spectroscopy-based metabolite profiling analysis and statistical analysis methods were used. Among the identified metabolites, six of them were found at significantly higher levels in dogs with TCC compared to control dogs, i.e., urea, choline, methylguanidine, citrate, acetone and β-hydroxybutyrate. These markers revealed good sensitivity to predict the healthy control and disease samples, suggesting a potential of urine metabolic profiling for early detection of bladder cancer [55]. Further studies involving larger cohorts will be needed to determine the accuracy and clinical usefulness of this approach as a screening, diagnostic or disease monitoring tool for canine TCC.

#### 3.3.11. Lipidomics

In one study, cancerous and noncancerous tissues were analysed for potential differences in their lipid profiles. Imaging analysis of tissue sections was performed, by using desorption electrospray ionisation mass spectrometry (DESI-MS). Differentiation between tissues was made using multiple marker lipids and free fatty acids. Samples included tissue sections from canine spontaneous invasive TCC of the urinary bladder, a cutaneous TCC metastasis sample and matched adjacent noncancerous tissues (*n* = 4). The obtained imaging mass spectrometry data were subjected to statistical analysis to determine whether the results correlated with those obtained from H&E staining, and with DESI-MS images of specific lipids [85]. 

Results indicated different lipid distributions between healthy and diseased tissues, concerning glycerophospholipids, sphingolipids and free fatty acids. The imaging technique enabled the distinction of canine cancerous bladder tissue and cutaneous metastasis from noncancerous canine bladder tissue samples. The obtained images agreed with those generated by DESI-MS and with H&E-stained tissue. The study concluded that DESI-MS imaging could be useful in diagnosing TCC by using a multimarker approach based on the lipid profiles and intensities of tissue samples. Further studies are required with larger populations and additional control groups, i.e., with other lower urinary diseases [85]. 

Lipid profiles were also assessed in urine from dogs with TCC, by using liquid chromatography-mass spectrometry (LC-MS). Urine samples from dogs with TCC, dogs with UTI and healthy dogs were analysed for potential differences in their lipid profiles (*n* = 15). In this study, 208 lipids were identified, belonging to several lipid families. Unique lipid profiles were found among the three cohorts, and specific statistical analyses allowed their differentiation. However, concentrations of the specific lipids could not be determined, and thus the study did not conclude which lipid families were up- or down-regulated in the urine samples of TCC patients compared to the other groups. Nevertheless, the study presented a foundation for further research on urinary lipids as potential biomarkers for TCC [86].

#### 3.3.12. Survivin

Survivin is an apoptosis-inhibiting protein expressed in both cancerous and noncancerous tissues. Expression of this protein was characterised by immunohistochemistry (IHC) in urinary bladder tissues from dogs with TCC, cystitis and dogs with normal urinary bladders. Cytoplasmic survivin was detected in all groups: 8% of cystitis tissues (2/24), 17% of TCC tissues (7/41) and 37% of normal tissues (17/46). However, significant differences were only seen between cystitis and normal groups. On the other hand, nuclear survivin was not detected in any of the normal bladder tissues (0/46), whereas 50% of cystitis tissues (12/24) and 68% of TCC tissues (28/41) were immunoreactive. The proportions of both TCC and cystitis positive specimens were statistically different compared to the normal group. However, TCC and cystitis tissues revealed comparable results. Furthermore, 57% of cystitic tissues (4/7) were positive for survivin mRNA, as well as 100% of TCC tissues (6/6) and 57% of normal tissues (11/22), even though these results were statistically comparable. It was hypothesised that this differential distribution of surviving within the cell in both cystitic and TCC tissues compared to normal tissues could be related to different functions of this protein depending on its location and that survivin could be implicated in tumour development in hyperplastic or inflamed tissues. Although survivin may not serve as a specific diagnostic marker for TCC of the urinary bladder in dogs, nuclear survivin could be detected in dogs with TCC or cystitis and not in normal dogs. Additional research is necessary to investigate whether nuclear survivin, particularly, can be involved in the development or progression of TCC and the possibility of nuclear survivin to be an early marker of bladder tumours and potential therapeutic target [87].

#### 3.3.13. EGFR

Epidermal growth factor receptor (EGFR) is a receptor tyrosine kinase of the ErbB family (which also includes HER2, HER3 and HER4), and its overexpression has been reported in several human and canine tumours. EFGR protein expression was evaluated by IHC in tissues from dogs with TCC (*n* = 25), polypoid cystitis (*n* = 5) and normal healthy bladders (*n* = 5), and specimens were divided into two groups depending on their staining scores: low-expression group and high-expression group. MRNA expression levels were also determined by quantitative real-time PCR in TCC (*n* = 4) and normal bladder (*n* = 3) tissues. Protein levels of EGFR were significantly increased in TCC compared to normal bladder and to polypoid cystitis tissues, while no significant difference was found between normal urinary bladder and polypoid cystitis tissues. Moreover, high EGFR protein expression was significantly associated with TCC, with a sensitivity of 72% and specificity of 100%. TCC exhibited significantly higher mRNA levels than normal urinary bladder, positively correlating with protein levels. No significant association was observed between EGFR protein expression and malignant tumour behaviour (presence of vessel invasion or lymph node metastasis) or with survival time in canine TCC [88]. 

EGFR expression could be used as a marker to aid canine TCC diagnosis. It may improve the sensitivity of urine cytological diagnosis when provisional diagnosis is needed. Development of a qPCR-based method could also be useful for diagnosing this malignancy, as it represents a more sensitive analytical method than IHC, requiring small amounts of samples [88]. Further research is essential for clarifying the underlying mechanisms of EGFR overexpression and tumourigenesis in canine TCC.

#### 3.3.14. HER-2

HER-2 is another member of the ErbB family that plays a role in the control of epithelial cells growth and differentiation and has been found to be overexpressed in human and canine cancers. Protein expression if HER-2 was assessed by IHC in bladder tissues from canine TCC (*n* = 23, of which 20 invasive TCC and 3 in situ papillary TCC) and compared with non-neoplastic canine urothelium (*n* = 5). Positivity to HER-2 was observed in 56% (13/23) of TCC specimens, and all control cases were considered negative. The receptor was found to be significantly overexpressed in TCC compared to non-neoplastic specimens, and expression did not statistically differ between invasive and in situ TCC [89]. HER-2 is the gene product of ERBB2, which was also found to be overexpressed in canine invasive TCC [61]. These results support the need for additional studies to further investigate the role of HER-2 as a potential marker of malignancy and therapeutic target in canine TCC [89].

#### 3.3.15. VEGFR2, PDGFR-β, c-KIT

Vascular endothelial growth factor receptor 2 (VEGFR2), platelet-derived growth factor receptor beta (PDGFR-β) and v-kit cellular homologue (c-KIT) are receptor tyrosine kinases (RTK), whose expression has been reported in multiple canine tumours. Toceranib phosphate is a receptor tyrosine kinase inhibitor that exhibits activity against these RTK and has been used anecdotally to treat canine TCC [90]. 

In one study, expression of these RTK was evaluated by IHC in bladder tissue samples from dogs diagnosed with TCC (*n* = 30), cystitis (*n* = 10) and in normal urinary bladder (*n* = 10). All TCC samples stained positive for PDGFR-β and for VEGFR2. The number of TCC samples that expressed PDGFR-β was significantly different from that of cystitis and normal bladder samples, with a more intense and diffuse staining in tumour cells, while no differences were found between normal bladder and cystitis groups. Regarding intensity scores or staining distribution for VEGFR2, no significant differences were observed between the three groups. However, most TCC samples exhibited intense cytoplasmatic staining for this RTK in >50% of tumour cells. Although 11/36 (36.7%) tumour samples showed positive staining for c-KIT, in most of them only minimal staining was noted (<1% of cells). No positive staining was observed in non-neoplastic tissues, and neither were there significant differences between the three groups [90]. Based on those findings, it was suggested that PDGFR-β could play a role in canine TCC tumourigenesis and that PDGFR-β and VEGFR2 might be involved in mediating clinical response of TCC to toceranib but that this would be unlikely for c-KIT [90].

Korec et al. (2021) confirmed the presence of these markers in canine TCC cell lines and tumour samples, and further supported the variable patterns of expression. However, activation of these RTKs was only present in a small subset of cells, which suggests that signalling through these pathways would less likely contribute to the aggressiveness of urothelial cancer cells [91].

Further studies are needed to investigate the presence of mutations associated with these receptors and to clarify the role of these RTKs in tumourigenesis, response to therapy and clinical outcome in dogs with TCC.

#### 3.3.16. Granzyme B, CD3

Infiltrates of immune cells can often be found in tumour tissues, and different types of infiltrating immune cells (e.g., tumour infiltrating lymphocytes) may affect tumour progression in different ways. IHC was used to investigate the localisation and number of CD3 (a T-cell maker) or granzyme B-positive cells in tissues from canine TCC and normal urinary bladder (*n* = 32 and *n* = 10, respectively). Granzyme B is a serine protease found in cytotoxic granules of cytotoxic T lymphocytes and NK cells that plays an important role in antitumour immunity. Both CD3 and granzyme B-positive cells were significantly increased in cancerous tissues compared with normal controls. The number of granzyme B^+^ cells was associated with favourable prognosis, while this was not noted for CD3^+^ cells. Primary tumour stage (WHO classification) was significantly associated with the overall survival. Additionally, no correlation was found between the number of CD3 and granzyme B-positive cells in TCC lesions and the primary tumour stage. It was therefore suggested that the presence of granzyme B^+^ tumour-infiltrating cells might be an independent prognostic factor in dogs affected with this malignancy. Granzyme B^+^ tumour-infiltrating cells could be involved in the inhibition of tumour progression in canine TCC. Additional studies are needed to identify and clarify the role of specific subsets of tumour-infiltrating immune cells in canine TCC and their potential association with prognosis [95].

#### 3.3.17. P63, Ki67, β-Catenin

The potential usefulness of proteins p63, Ki67 and β-catenin as clinical markers for predicting biological behaviour and prognosis was investigated in canine TCC [96]. It is thought that p63 plays an important role in cellular development and differentiation of stratified epithelia in skin, prostate gland, mammary gland and urinary bladder [96,97,98,99]. In humans, loss of p63 expression has been associated with tumourigenesis and malignancy [100,101]. Ki67 is a nuclear protein expressed by proliferating cells, while membranous β-catenin is involved in cellular adherence, and a reduced expression of this protein has been linked to progression and poor prognosis in human urothelial carcinoma [96].

Expression of these proteins was evaluated by IHC in tissue samples from dogs with TCC (*n* = 25), polypoid cystitis (*n* = 5) and in normal urinary bladder (*n* = 5). In TCC samples, staining scores for p63 and β-catenin were significantly lower than in polypoid cystitis and in normal urinary bladder, whereas significantly higher staining scores for Ki67 were noted in TCC samples compared to both other tissue groups. Comparable results were observed for all proteins between cystitis and normal urinary bladder tissues [96]. 

Furthermore, low p63 expression in TCC was significantly related with the presence of vessel invasion and metastasis, and with short survival time [96]. Similar findings and correlations were observed with expression of ΔNp63, an isoform of p53 [102]. Based on this evidence, the authors suggested that p63 could be used as a clinical marker for diagnosing and predicting prognosis in canine TCC. Additional studies are needed, namely on investigating molecular features, such as p63 gene expression [96].

#### 3.3.18. UIII, CK 7, CK 20, COX-2, Activated Caspase 3, GATA-3

Uroplakin III (UP III) belongs to a group of membrane-associated proteins expressed by urothelial cells [103]. It is the most common marker of urothelial differentiation used in dogs [104]. Cytokeratin 7 (CK 7) and cytokeratin 20 (CK 20) are cytokeratins expressed by simple epithelium, as well as urothelial cells, and have been used for characterising urothelial tumours.

The expressions of UP III, CK 7 and CK 20 were (separately) characterised by IHC in tissues from canine urinary bladder tumours (*n* = 72) and from normal urinary bladders. Tumours from the urinary bladder comprised TCC (*n* = 60 total, including 5 metastatic cases and 2 TCC from the ureter and renal pelvis), transitional cell papillomas, rhabdomyosarcomas, squamous cell carcinoma, lymphosarcoma and spindle cell sarcoma. Additionally, 285 canine tumours/lesions not associated with the urinary bladder were evaluated for UP III expression, as were multiple other normal tissues. UP III was identified in superficial (umbrella) cells and some intermediate cells of the normal urinary bladder, as well as in the majority of TCCs (91% of primary (50/55) and 80% of metastatic TCCs (4/5)) and in all transitional cell papillomas (7/7). The remaining tumours of the urinary bladder, as well as non-urothelial normal and neoplastic tissues, were negative for UP III. Staining for CK 7 was detected in 53 out of 54 TCC (98%), in all 5 metastatic TCC and all 7 transitional cell papillomas. Concerning CK 20, staining was observed in 37 out of 54 TCC, 1 metastatic TCC and in 1 out of 7 transitional cell papillomas. Concurrent expression (detection of several antigens in the same tumour) of UP III, CK 7 and CK 20 was identified in 67% (36) of primary TCC and in 1 metastatic TCC. The only anaplastic TCC evaluated was negative for both CK 7, CK 20 and UP III, although the adjacent normal urothelium was positive for UP III [104]. 

The authors suggested that the pattern of staining for UP III could help distinguish between carcinoma in situ and invasive TCCs. UP III was shown as a specific and sensitive marker of canine transitional epithelial neoplasms. Even though CK 7 was more sensitive than UP III for canine TCC, CK 7 is expressed in several non-urothelial tumours and also in normal tissues, as is CK 20. The authors considered UP III as the marker of choice in canine urothelial neoplasms and that CK 7 should be used for tumours negative for UP III but suspected of being TCC. Negative results could be obtained with anaplastic tumours. CK 20 alone did not prove to be useful for diagnosis of urothelial tumours [104].

In a subsequent study, Sledge and co-authors (2015) [105] evaluated the expression of UP III, CK 7 and also COX-2 and activated caspase 3 in canine urothelial lesions. Cycloocygenase-2 (COX-2) and prostaglandin E2 have been implicated in carcinogenesis at various levels. COX-2 has been found to be expressed in canine TCCs but not by normal urothelium of the urinary bladder. Detection of activated caspase 3 expression by IHC has been used to evaluate apoptotic rate, and this protein has been suggested to have prognostic significance in human urinary bladder cancers [105]. Expression of aforementioned markers was evaluated by IHC in canine urothelial lesions and compared with each lesion’s classification and grade. Proliferative urothelial lesions of the urinary bladder from dogs (*n* = 99) were examined, including non-neoplastic lesions (urothelial polyps and polypoid cystitis), low-grade neoplasms (urothelial papillomas, papillary urothelial neoplasms of low malignant potential and grade 1 urothelial carcinomas) and grade 2 and 3 urothelial carcinomas [105]. 

Results showed overall strong differences in the patterns of UP III, CK 7 and COX-2 expression in canine urothelial proliferative lesions of the urinary bladder. Furthermore, significant associations were identified between tumour classification and overall UP III pattern, loss of UP III expression, overall CK 7 pattern and COX-2 pattern, as well as between depth of neoplastic cell infiltration into the urinary bladder wall and overall UP III pattern, loss of UP III, overall CK 7 pattern, loss of CK 7 expression and COX-2 pattern. Regarding the expression pattern of activated caspase 3, no significant association was observed with tumour classification, grade or infiltration. Based on these findings, it was hypothesised that loss of UP III and CK 7 in urothelial carcinomas could suggest a lack of differentiation or epithelial-mesenchymal transition favouring infiltration. Moreover, some urothelial carcinomas might not be positively labelled when using UP III and CK 7 as diagnostic markers [105]. 

It should be noted that UP III is not a specific marker of TCC itself, since it does not differentiate neoplastic from non-neoplastic lesions. However, it can be useful, for instance, to rule in TCC in a biopsy from a tumour of unknown origin and to identify metastatic carcinomas on the skin [2]. 

More recently, the expression of COX-2 was studied by IHC in TCC biopsies from 65 dogs from different breeds (both terriers and non-terriers) [44]. Samples were graded histologically into low- and high-grade. Both neoplastic and inflammatory cells expressed this marker, whilst it was not detected in normal transitional cell epithelium. The intensity of COX-2 expression was highly variable within neoplastic specimens, but it did not correlate with grading, which is different to what has been observed in humans. Samples were also tested for the *BRAF* mutation. A positive correlation was found between presence of the mutation and the intensity of expression of COX-2 in TCC from non-terrier breeds. This was not as clear for the terriers, although many fewer cases of this breed were analysed, so a significant result would not be completely ruled out. More studies would be encouraged to further determine the clinical and prognostic relevance of COX-2 expression, both on its own and in association with *BRAF* mutation and breed [44].

GATA-Binding Protein 3 (GATA3) is a zinc finger transcription factor that has been used in human medicine as a diagnostic and prognostic marker of urothelial carcinoma [117,118,119]. In a review article, Knapp et al., 2014 [114] showed the expression of GATA3 in a canine TCC bladder tissue sample. To the best of our knowledge, no other studies have yet looked at its role in canine TCC.

Further studies on the expression of these markers, especially concerning their association with grading and prognostic evaluation, are encouraged. 

## 4. Therapies for UC

Nowadays, a wide range of treatments have been studied that could lead to remission of TCC or provide stable disease for several months. Therapeutic options for TCC include surgery, radiation therapy, systemic and localised medical therapy, and combinations of these [1].

### 4.1. Surgical Approaches

Surgery may be performed for collecting tissue samples for diagnosis and for tumour removal (if lesions are located outside of the trigone region). However, complete surgical excision is often not feasible due to the trigonal location of the tumour and/or multifocal distribution in the bladder, urethral involvement and presence of metastases [1,120]. In cases where excision is possible (e.g., tumours located in the apical region of the bladder), surgery may prolong survival, and chemotherapy following excision should be considered [121,122,123]. Caution should be taken during surgery to avoid cancer seeding along the surgery site and the abdominal wall [1,120].

It is noteworthy that incontinence is an expected outcome of total cystectomy, which is another reason why this procedure is infrequently carried out in dogs [124].

Surgical procedures may also be used for the management/palliation of urethral obstruction secondary to TCC, to maintain or restore urine flow. For this purpose, cystotomy tubes or catheters can be placed to allow bypassing urethral obstruction. Moreover, ureteral or urethral stents can be placed surgically or by using less invasive techniques [1,125,126,127].

Ureterocolonic anastomosis, a technique that consists of transecting the ureters and anastomosing them to the colon, has been reported in dogs with TCC, along with the complete excision of the bladder and other affected structures. The technique allowed diversion of urine into the colon with maintenance of continence by the anus. However, several neurological and gastrointestinal complications developed over time, with limited survival, and therefore the procedure has not been recommended [120,128]. More recently, a procedure involving total cystectomy and urinary diversion to the prepuce or vagina (ureteropreputial or ureterovaginal anastomosis) in dogs with TCC of the trigonal area was associated with fewer gastrointestinal and neurological complications, although high morbidity and complication rates were still seen compared with techniques used in humans [124]. Cutaneous ureterostomy with radical cystectomy has also been reported in dogs with invasive trigonal TCC, with both ureters being transected and anastomosed to the ventral abdominal skin. The procedure was viable, and results suggested survival benefits compared to other methods, even though further studies are required to evaluate the potential survival benefits in a larger population [129]. Urinary incontinence is invariably associated with these two latter procedures, requiring owner compliance with life-long diaper changes and hygiene protocols. Therefore, these options may be contraindicated in dogs that are intolerant of direct owner handling [124,129]. 

Recently, a laparoscopic technique was used for the removal of a TCC in the distal part of the urethra in a female dog. A pre-pubic urethrostomy and laparoscopic removal was performed. Patient remained clinically stable with no urinary incontinence for 2.5 months. The use of these minimally invasive procedures is therefore promising in some cases for the complete removal of urethral tumours where possible and, in inoperable cases, as a palliative alternative [130]. 

Liptak et al. (2004) [131] reported the use of electrosurgical transurethral resection in dogs with neoplastic obstruction of the lower urinary tract. Despite showing promising results for male dogs with prostatic carcinoma, the procedure was not recommended in female dogs with urethral TCC due to high complication rate, including urethral perforation. Laser-based techniques have also been described for the ablation of TCC and/or to relieve urethral obstruction caused by the tumour, combined with other therapeutic options [132,133]. However, further studies are necessary to clarify their potential benefits.

### 4.2. Radiation Therapy

The use of radiation therapy (intraoperative and/or external beam) has been reported in a few studies as a first-line or rescue therapy, occasionally combined with surgery, systemic chemotherapy or NSAIDs. The approach has been associated with several complications and is not routinely used in the management of TCC. One of the challenges is related to variations in size, position and shape of the bladder and surrounding structures throughout treatment, with surrounding normal tissues often being irradiated within the pelvic region. However, more recent protocols have shown improved tolerability, encouraging further studies [134,135,136,137,138]. The contribution of radiotherapy to antitumour activity in multimodal approaches requires better clarification, as well as its benefits compared to other therapies.

### 4.3. Chemotherapy/Systemic Medical Therapy

The current mainstay of treatment for canine TCC includes systemic medical therapy, usually with chemotherapy, non-steroidal anti-inflammatory drugs (NSAIDs) or combinations of these [1,123]. Medical therapy is not typically curative. However, several different agents can produce remission or stable disease, and most of them are well tolerated [1]. 

Numerous chemotherapeutic drugs and protocols have been reported for canine TCC, either as single agents or in combination therapies, including the genotoxic agents carboplatin [139,140], cisplatin [141,142], doxorubicin [122], gemcitabine [143], mitoxantrone [132,134,140,144], vinblastine [145,146], vinorelbine [147] and metronomic chlorambucil [148].

Concerning NSAIDs, nonselective cyclooxygenase (COX) inhibitors and COX-2 specific inhibitors have been used for their antitumour activity [142,149]. Piroxicam [77,122,132,134,140,143,144,150] is generally preferred as the first-line NSAID, but deracoxib [151] and firocoxib [142] have also been studied for the treatment of canine TCC [123,142,150,151]. The most frequently used chemotherapy protocol consists of a combination of mitoxantrone and piroxicam [1,140,144].

Adverse effects of NSAIDs include both nephro- and gastro-intestinal (GI) toxicity. COX-2 selective inhibitors are known to cause fewer GI effects; however, the effectiveness of these ones compared to nonselective COX inhibitors is still unclear. Assessment of renal and liver function prior to chemotherapy is recommended, as well as routine monitoring of these parameters and of GI signs during the course of treatment. Drugs such as omeprazole or famotidine could be used to reduce the risk of GI effects [1,123]. 

As mentioned in Section 3.3.18, COX-2 has been found to be expressed in canine TCC. However, it is still unclear whether expression of this molecule could be useful as a predictive factor for the response to COX-2 inhibitors. 

Concerning cytotoxic chemotherapy, the risks and intensity of side effects vary with each drug, including GI signs and bone marrow suppression, likewise requiring frequent monitoring of overall health parameters. Overall, the majority of dogs (*circa* 80–85%) do tolerate these treatments with a good quality of life [1,123].

### 4.4. Localised Therapies

Regarding localised treatments, intravesical therapy is frequently used in humans with superficial TCC and has been investigated in dogs too [120]. 

Intravesical administration of mitomycin C has been carried out in dogs with naturally occurring TCC as a chemotherapeutic agent, showing promising antitumour activity. Severe adverse events were occasionally observed, possibly due to systemic absorption of the drug, thus requiring further investigation [152].

Bacillus Calmette-Guérin (BCG) has successfully been used in humans for the treatment of non-muscle invasive bladder cancer [153]. In one study with healthy dogs, published in 1975, severe local inflammatory reactions were caused after BCG was instilled in their bladders [154]. No recent data on clinical trials in dogs with TCC were found, and there are concerns about the risk of systemic absorption, among others.

IL-2 is a cytokine that is involved in several immunological processes. This protein is secreted by different types of immune cells and binds to IL-2 receptors, which are also expressed by multiple immune cell populations. Within its numerous functions, IL-2 is capable of recruiting cytotoxic T-lymphocytes selectively to tumours [155,156]. This cytokine has been used in cancer immunotherapy in both human and veterinary medicine, mostly via its local application into tumour lesions [156]. When locally applied, IL-2 is usually much more effective and causes fewer side effects compared with the systemic route [157]. Local administration of IL-2 induces vascular leakage and tumour necrosis, with subsequent stimulation of an immune response. Time required until tumour regression varies with the degree of tumour vascularisation, ranging generally from a week to several months [156]. 

The influence of intralesional treatment with interleukin-2 (IL-2) on the clinical course and tumour progression of canine TCC was evaluated in a retrospective clinical study. Twenty-five dogs with advanced TCC of the urinary bladder and/or urethra, in which curative surgery was unfeasible, were treated. This was achieved via either transabdominal ultrasound-guided intralesional injection of IL-2, endoscopically/transrectally assisted or by IL-2 injection into the tumour bed after cytoreductive (palliative) surgical tumour resection. Additionally, all dogs received long-term NSAIDs, and a chemotherapeutic agent was used in two dogs. Adverse effects associated with intralesional IL-2 treatment were not observed, and at the time of re-examination (which varied individually), 17 dogs showed marked clinical improvement and regression of tumour size, and 4 dogs were in complete remission. IL-2 intralesional application was considered a safe and minimally invasive palliative treatment modality for canine advanced TCC in the impossibility of a surgical cure. Further prospective studies are warranted for clarifying the efficacy of intralesional IL-2 treatment in dogs with TCC [156].

Photodynamic therapy has also been studied in healthy dogs and dogs with spontaneous TCC by orally administering 5-aminolevulinic acid (ALA), which is converted to a photosensitiser metabolite, protoporphyrin IX (PpIX). In healthy dogs, fluorescence of PpIX was confined to the mucosa and submucosa layers of the urinary bladder and substantially increased in the former. In diseased dogs, the observed long-term responses suggested the usefulness of this approach for treatment of canine TCC, requiring further research [158,159].

Nanoparticles carrying chemotherapeutic agents have been used experimentally for drug delivery to canine bladder TCC cells [160,161,162,163,164,165]. Gelatin nanoparticles containing paclitaxel have been delivered intravesically to dogs with TCC [166]. Furthermore, targeting nanomicelles coated with the ligand PLZ4, a peptide that specifically binds to both human and dog bladder TCC cells, were able to efficiently deliver chemotherapeutic drugs (daunorubicin or paclitaxel) and imaging agents into the tumour site in vivo, in a mouse model carrying canine bladder cancer xenografts [160,161,162]. More recently, Lin and co-workers (2016) developed a multifunctional platform of nanoporphyrins that was also coated with PLZ4 (PNPs), which are nanoparticles with the ability to emit fluorescent signals/heat/reactive oxygen species when illuminated with specific light. Doxorubicin-loaded PNPs were tested in vitro in both human bladder cancer cells and normal canine urothelial cells, and in vivo in a mouse model. The platform selectively targeted tumour cells for photodynamic diagnosis and was also effective against bladder cancer, integrating three therapeutic modalities in a single procedure (photodynamic, photothermal and chemotherapeutic), with either intravesical or systemic administration [164]. In the future, investigation is warranted on the efficacy and toxicity of these nanotechnology-based approaches to translate them into both veterinary and human clinical application.

Another targeted therapy that is being investigated in dogs is a treatment targeting folate (vitamin B9) receptors, which is based on the high uptake of folate and folate drug conjugates into certain cancers compared with normal tissues [114]. In one study evaluating canine and human tissues from invasive urothelial carcinomas, folate receptors were detected in most primary UC tissues and nodal and lung metastases from dogs, and folate uptake was detected in primary and metastatic lesions. Dogs with folate-receptors-positive invasive urothelial carcinoma were treated with a folate-targeted vinblastine compound in a dose escalation study, showing promising initial results with tumour responses, including partial remission and stable disease, and further study is ongoing [167].

### 4.5. Epigenetic-Based Therapies

Therapies targeted at epigenetic changes are also being developed. 

Aberrant methylation in the promoter region of tumour suppressor genes, causing gene silencing, is an example of the epigenetic events that can lead to cancer development and progression in the absence of DNA mutations [168]. Several DNA methylation markers have been studied, with growing evidence supporting their usefulness for the diagnosis, prognosis and treatment of human bladder cancer. Aberrant DNA methylation in cancer-related genes has been reported in human TCC, cell lines and urine sediments [169,170,171,172]. 

DNA methyltransferase 1 (DNMT1), an important enzyme in DNA methylation, has been found to be overexpressed in both human and canine TCC and was identified as a potential target in the treatment of TCC, but data are lacking concerning its potential applications in canine TCC [173,174]. DNMT1 inhibitors have shown antiproliferative effects both in vitro and in vivo [174,175]. In a preclinical phase I trial, dogs with naturally occurring invasive UC, used as a model for human UC, were treated with 5-azacitidine subcutaneously, and this compound exhibited a promising clinical/antitumour activity, encouraging further studies [175]. Zebulatine, an orally bioavailable agent that also has demethylating activity with a similar mechanism of action to that of 5-azacitidine, was studied in three laboratory dogs and three tumour-bearing dogs, two of them with invasive TCC. In three laboratory dogs, the treatment resulted in some severe but reversible adverse effects, while no appreciable toxicity was observed in the tumour-bearing dogs, with those with invasive TCC showing stable disease. At the time of writing, a subsequent dose escalating trial is ongoing in dogs with invasive TCC [176].

Another example of an epigenetic change is histone acetylation. Histone deacetylases (HDAC) have been suggested to be overexpressed in both human and canine tumours. Excessive activity of HDAC could facilitate the deacetylation of histones, causing down-regulation of the expression of tumour suppressor genes. Molecules that inhibit the activity of HDAC have been evaluated as a possible antitumour therapy in both humans and dogs (reviewed by Xavier et al., 2020 and Goutas et al., 2021 [177,178]). It is thought that HDAC inhibitors (HDACi) induce the acetylation of deacetylated histones, thereby promoting the expression of tumour suppressor genes, which could result in an antitumour effect [179]. 

Vorinostat, a HDACi, was found to have an antitumour effect on canine UC cell lines, both in vitro, and in vivo in a xenograft mouse model. It inhibited cell growth and induced G0/G1 cell cycle arrest. This HDACi induced histone acetylation and the expression of cell-cycle related molecules [179]. Moreover, histone deacetylation was aberrantly observed in canine UC tissues compared to normal samples, suggesting that epigenetic dysregulation may play a role in canine UC progression, such that lower levels of deacetylation were related to a poor prognosis [179]. More studies will be required to further investigate these findings and the role of HDACi as a potential therapy for UC.

### 4.6. Other Emerging Targeted Therapies

Toceranib phosphate is a RTK inhibitor that has been used anecdotally to treat TCC in dogs. This small-molecule inhibitor targets c-KIT, VEGFR2 and PDGFR-β, among others. It is approved for the treatment of cutaneous mast cell tumours in dogs, and it is also active against several other cancer types. As mentioned in Section 3.3.15., it was recently suggested that PDGFR-β could play a role in canine TCC tumourigenesis and that PDGFR-β and VEGFR2 could be involved in mediating clinical response of TCC to toceranib. However, the mechanisms by which this could take place are not entirely understood [90,91]. 

In a phase I clinical trial with dogs with spontaneous malignancies, treatment with toceranib (SU11654) resulted in stable disease in three out of four dogs with TCC of the bladder [92]. A pilot study investigated the biological activity of the combination of toceranib and vinblastine for treatment of canine TCC of the bladder, but response to therapy was not improved when compared to treatment with single-agent vinblastine. However, lower doses of vinblastine were used in the combination protocol, sample size was limited, and a prospective control group was also lacking [93].

In a retrospective study, 37 dogs were treated for TCC with toceranib as a second- or third-line therapy. Of the 15 dogs in whom treatment response was evaluated, 13 were concomitantly treated with an NSAID. A partial response was achieved in 1 of the patients, while 12 (80%) dogs had stable disease for a median of around 128 days (34–310). The drug was overall well tolerated, although azotaemia progressed in over half of the patients [94].

In a more recent study with canine TCC cell lines that expressed these markers, treatment with toceranib had no significant effect on cell proliferation [91]. 

It is possible that stable disease, rather than tumour regression, is related to the mechanism of action of toceranib, by inhibiting growth factor signalling, and not directly damaging tumoural DNA [94]. Further prospective studies with larger samples and comparing different treatments are encouraged. 

The identification of the *BRAF^V595E^* mutation in canine TCC and its high prevalence pointed to the possibility of targeting the BRAF/MAPK pathway via a therapeutic approach in those tumours carrying the mutation. In humans, several drugs, such as vemurafenib, a kinase inhibitor, have been developed to selectively target the *BRAF^V600E^* mutation in different malignancies, with high effectiveness. However, innate or acquired resistance to BRAF inhibitors can occur in some *BRAF^V600E^*-positive cancers, and the presence of the mutation does not always correlate with clinical response to BRAF inhibitors [58,59,180]. 

Vemurafenib was studied in canine TCC cells and showed anti-proliferative effects [58]. In a subsequent phase I/II clinical trial, this drug was tested in dogs with naturally occurring invasive TCC, which were positive for the *BRAF^V595E^* mutation. In 9 of the 24 dogs (38%), partial remission was achieved, with a median progression-free interval of 181 days (53 to 608 days). Anorexia was the most common side effect. Similar to what has been observed in humans treated with this drug, new tumours developed in some of the patients (cutaneous SCC, papillomas). Resistance to therapy also occurred in some animals over time [65].

Sorafenib is another kinase inhibitor that targets multiple kinases, including RAF, VEGFRs and PDGFR-β, with anti-proliferative and anti-angiogenic activities. It is approved for the treatment of various cancers in humans, and it has been studied in veterinary medicine too, being well tolerated by dogs in preliminary studies [66,67,110,181].

In in vitro studies with established TCC cell lines derived from dogs harbouring the mutation, this drug inhibited the RAF/MAPK pathway and induced apoptosis. These effects were more pronounced with sorafenib than with vemurafenib. The reasons for these differences were not fully clear, so future studies would be needed [67].

Furthermore, sorafenib was used in a dog with metastatic TCC of the urethra, in whom complete surgical excision was not achievable. Tumour tissue was *BRAF^V595E^*-positive and showed overexpression of VEFGR. After unsuccessful response to chemotherapy with mitoxantrone, sorafenib was trialled in association with piroxicam. To monitor treatment response, *BRAF* mutation levels were measured in ctDNA obtained from the patient’s serial plasma samples. Genetic sequencing techniques were used. During treatment, levels of mutated *BRAF* varied; they tended to increase with the severity of the clinical signs (dysuria) and decrease when clinical signs improved after sorafenib was escalated to the maximum tolerated oral dose. Dysuria was well managed, and a decrease in thickness of urethral wall was seen. The patient remained stable for over 3 months of treatment, with few side effects [110]. 

The effect of sorafenib on VEGFR expression, as well as the effects of sorafenib alone versus the combined treatment, would require further study, but overall, these results were promising, and further clinical research is encouraged.

Additional in vitro and in vivo evaluations of the effects of therapies targeted at these and other canine TCC markers with potential applications in a clinical setting are underway. 

## 5. Conclusions

The last few years have witnessed great progress concerning our understanding of bladder cancer. In dogs, bladder cancer consists mainly of TCC with poorly characterised aetiology, a complex molecular landscape, heterogeneous morphology and diverse biological behaviour, requiring effective prognostic and therapeutic markers. In contrast, cattle, who also show a significant incidence of bladder cancer, are affected by well-defined etiologic factors that deregulate specific signalling pathways. 

Histological analysis of canine TCC is essential but insufficient to determine patient prognosis and reach a tailored therapeutic approach. In this context, the recent development and validation of TCC molecular markers is of great importance for scientists and clinicians alike. Somatic and hereditary BRAF mutations received much attention and can now be detected via multiple types of tests, sometimes in useful combinations with CNA tests. Urine-based tests for detecting BRAF may allow the early detection of post-treatment relapse. Other urine-based tests (e.g., for bFGF and calgranulins levels) have also found interesting clinical applications. A number of tissue-based markers have also been put forward (e.g., immunohistochemical detection of COX-2), but their use to predict prognosis or response to therapy is still under study. Additional work on blood-based liquid biopsies is expected in the coming years, aiming to match similar developments obtained for human patients. Minimally invasive techniques have proved valuable and more effective as biomarkers than tissue-based approaches. A more detailed knowledge of the molecular signalling pathways involved in canine TCC will help design more effective targeted therapies and new tests with enhanced predictive value for canine TCC patients.

## Figures and Tables

**Table 1:** Primary bladder tumours in dogs, types and percentages (the most common tumours reported are shown; adapted from Meuten and Meuten, 2016 [2]).

Primary Canine Urinary Bladder Tumours			
**Epithelial**			
**Malignant**	(%)	**Benign**	(%)
Transitional cell carcinoma	75–90	Papilloma	2
Undifferentiated carcinoma	6		
Adenocarcinoma	4	Adenoma	0.2
Squamous cell carcinoma	3		
**Mesenchymal**			
**Malignant**		**Benign**	
Leiomyosarcoma	2	Leiomyoma	2
Sarcoma	1.5		
Rhabdomyosarcoma	1.3		
Haemangiosarcoma	1	Haemangioma	0.2
Fibrosarcoma	1	Fibroma	1

**Table 2:** Classification of canine TCC subtypes based on growth pattern (adapted from Meuten and Meuten, 2016 [2]).

Canine Transitional Cell Carcinoma Classification
**Papillary infiltrating**
Often multiple and may cover large regions of the mucosa. Form papillary or exophytic growths that project into the lumen of the bladder. Invade the stalk and wall of the bladder, lamina propria, and muscle layers and may be transmural. Mild to marked cellular atypia. Likely to metastasise.
**Papillary non-infiltrating**
Do not invade the stroma of their own stalk, do not go beyond the lamina propria, so unlikely to metastasise. Differentiation from papilloma is subjective and based on criteria such as overall size, cellular atypia, small branches off the main lesion, among others. Non-invasive tumours may be adjacent to invasive TCC, and additional sections should be searched for invasion.
**Non-papillary infiltrating**
Form plaques and flat nodules, which can cover large regions of the mucosa. Surfaces are often ulcerated, tumour infiltrates into muscle layers, so high tendency to metastasise. Marked histological and cytological variability.
**Non-papillary non-infiltrating**
Rare. Additionally, defined as carcinoma in situ; confined to the epithelium and do not form papillae. Neoplastic epithelium more intensely eosinophilic than non-neoplastic cells; cells may be dysplastic to mildly anaplastic. Loss of intercellular cohesion. Usually located adjacent to invasive carcinoma; if seen, additional section analysis recommended to look for invasion.

**Table 3:** Current and potential markers for clinical applications in canine transitional cell carcinoma.

Biomarker	Sample	Method	Diagnostic Utility, Commercial Availability	Utility as a Prognostic and/or Therapeutic Target	Power of the Test	
					Sensitivity	Specificity
***BRAF* mutation**	**Tissue, urine, blood** [58,59,60,61,62,63,64,65,66,67]	Determination of *cBRAF^V595E^* mutation status in DNA retrieved from cells, using ddPCR analysis or other molecular methods.	Highly sensitive test for detecting TCC cells bearing the *BRAF* mutation. Could be used as a first, non-invasive screening test. Commercially available for dogs, for use in free-catch urine samples—CADET^®^ *BRAF* mutation detection assay. Provides qualitative results (positive vs. negative for V595E) and quantitative data of tumour-derived mutation load in urine DNA. Reported to detect TCC in free-catch urine samples up to several months before development of clinical signs. The test is not affected by the presence of blood or bacteria in the urine. ~20% of tumours of canine TCC and PC patients do not possess the mutation, which limits the sensitivity of the ddPCR assay to ~80%. A more recent test that detects chromosomal copy number variation can be added in *BRAF* mutation-negative patients, increasing combined sensitivity to ~95% (CADET^®^ *BRAF-PLUS).*	BRAF mutation was not a predictor for histological grade, nor for survival. Measuring levels of BRAF mutation in urine or blood samples may be useful for monitoring treatment response and relapse. Potential target for treatment.	67–88% (TCC, tissue) 83–100% (TCC, urine)	100% (TCC, tissue and urine)
**BTA**	**Urine** [68,69,70,71,72]	Rapid latex agglutination dipstick colorimetric test for qualitative detection of tumour analytes in urine. The test uses antibodies to detect a urinary bladder tumour-associated glycoprotein complex.	Useful as a screening test to rule out TCC, especially in dogs at high risk of developing TCC. False positive test results reported in dogs with non-neoplastic urinary tract disease, e.g., in the presence of significant glycosuria, proteinuria, and pyuria or haematuria. Presence of lower urinary tract malignant tumours other than TCC may yield positive results. Discrepancies with results may be observed over time, while reading the test. Commercially available—V-BTA Test. Results are either positive or negative. Not recommended as a confirmatory/definitive diagnostic test for urinary tract TCC in dogs, and should not be indiscriminately used in every patient presenting clinical signs of urinary tract disease.	N.A.	88–90%	35–41% in dogs with non-malignant urinary tract disease; 84–94% in healthy dogs or unhealthy dogs due to non-urinary tract diseases
**bFGF**	**Urine** [73,74,75,76,77,78]	ELISA urine test for human and canine bFGF. A quantitative sandwich enzyme immunoassay technique has also been developed using an antibody for canine bFGF.	Urine bFGF could be useful as a diagnostic tumour marker, helping to distinguish dogs with UTI from those with TCC. Commercially available (for research use, only): Quantikine^®^ HS ELISA, Human FGF basic Immunoassay, Canine BFGF ELISA Kit, Nori^®^ Canine FGF Basic ELISA Kit.	Quantification of urine bFGF could be useful as a non-invasive indicator of treatment response.	N.S.	N.S.
**Chromosomal CNAs**	**Tissue, urine** [39,79]	Assessment of urothelial cell ploidy/DNA copy number status in biopsy sections and in urine sediment by FISH.	Non-invasive method for canine TCC diagnosis. Potentially high-sensitivity and high-specificity FISH-based method/assay for the detection of canine TCC diagnosis utilising low-volume, free-catch urine specimens. Expensive and high effort method/labour intensive, expertise, time-consuming, increased cost, which may limit its application as routine diagnostics in a clinical environment. Not commercially available for canine TCC. Available for in vitro diagnostic use in human samples. A multicolour FISH-based assay for detection of aneuploidy for chromosomes 3, 7, 17, and loss of the 9p21 locus through FISH in urine specimens—UroVysion Bladder Cancer Kit.	N.A.	N.S.	N.S.
	**Tissue, urine** [55,64]	Multiplexed ddPCR assay for the detection and quantification of DNA copy number imbalances/changes characteristic to canine TCC.	Accurate, high-throughput method for evaluation of copy number changes in dogs with TCC. In this study, changes in copy number were not detected in 33% of urine DNA samples from dogs with TCC, which was probably due to the presence of inflammatory cells. Thus, additional techniques to improve sensitivity in those samples may be required. In such cases, FISH will still provide a more accurate evaluation. Commercially available for dogs, for use in free-catch urine samples: CADET^®^ *BRAF-PLUS*. Can be used in *BRAF* mutation-negative patients. Could be added to CADET^®^ *BRAF*, increasing combined sensitivity to ~95%.	N.A.	N.S.	N.S.
**Microsatellite instability**	**Urine** [72]	PCR study of a panel of 22 microsatellite DNA sequences from exfoliated urothelial cells and blood cells; comparison of microsatellites genotypes.	The technique added little value as a diagnostic test for TCC in dogs. High rate of false positives (32%, 12 of 38).	N.A.	55% (48% *)	68% (76%, vs. V-BTA)
					* When compared with results of V-BTA from the same study.	
**MicroRNAs**	**Tissue, cell lines** [56]	QPCR of specific miRNAs involved in the pathophysiology of TCC in humans.	MiR-34a, miR-16, miR-103b and miR-106b could be useful diagnostic biomarkers for the identification of dogs with TCC. More studies are required, with a larger sample.	N.A.	N.S.	N.S.
	**Blood, urine** [80]		MiR-103b and miR-16 are potential non-invasive diagnostic biomarkers for TCC; particularly for distinguishing LUTD and TCC in canine urine samples. Urine tests seem to be superior in distinguishing TCC from LUTD.	N.A.	N.A.	N.S.
**Telomerase**	**Canine TCC cell line, urine** [81,82]	PCR-based telomeric repeat amplification protocol for detection/measurement of telomerase activity.	Telomerase activity may be useful in diagnosing canine TCC in urine samples in a clinical context. Results of the assay are either telomerase-positive or telomerase-negative. Urine samples containing other telomerase-positive cells may yield false-positive results (e.g., presence of activated lymphocytes in dogs with bacterial cystitis). False-negative results may occur with unappropriated urine samples storage.	N.A.	91%	89%
					Diagnostic sensitivity/specificity of the TRAP assay applied to clinical canine urine samples.	
**Calgranulins**	**Urine** [44,83]	Species-specific radioimmunoassays to measure urine concentrations of canine calgranulins S100A8/A9 and S100A12.	Results presented as normalised to urine specific gravity levels (S100A8/A9_USG_) and as S100A8/A9-to-S100A12 ratio (UcalR). Provides quantitative results. S100A8/A9_USG_ could be a good a screening test for TCC/PC in dogs, especially in those where a UTI has been ruled out as a cause of clinical signs of lower urinary tract disease (due to a moderate rate of false positives observed for dogs ≥6 years of age with UTI). UcalR can help differentiate patients with a UTI from those with TCC/PC, even though a moderate false negative rate was seen in dogs ≥ 6 y.o. with a UTI. A combination of S100A8/A9_USG_ and uCalR improved diagnostic accuracy for the detection of canine TCC/PC. Test levels are not affected by haematuria.	N.A.	96% S100A8/A9_USG_ * 91% UcalR **	66% S100A8/A9_USG_ * 60% UcalR **
					* For detection of TCC/PC in dogs ≥ 6 y.o.; ** to distinguish dogs with TCC/PC from dogs with UTI in dogs ≥ 6 y.o.	
**Proteomics**	**Urine** [84]	Characterisation of the canine urinary proteome by using liquid chromatography tandem mass spectrometry and immunoblot.	A protein signature was identified, that could distinguish between healthy patients and those with TCC or UTIs. A statistical model using a biomarker multiplex for categorising samples as TCC or non-TCC was developed, predicting the presence of disease with 90% confidence. Potential relevance of the identified proteins as biomarkers for the diagnosis of TCC in dogs. Preliminary study, high-throughput technique. A more direct assay will be useful for clinical diagnosis.	N.A.	N.S.	N.S.
**Metabolomics**	**Urine** [55]	Nuclear magnetic resonance spectroscopy-based metabolite profiling analysis.	Six metabolites showed significantly higher levels in dogs with TCC compared to controls: urea, choline, methylguanidine, citrate, acetone and β-hydroxybutyrate. Good sensitivity to predict the healthy control and disease samples. Potential for early detection of bladder cancer. Preliminary study, high-throughput technique.	N.A.	86%	78%
**Lipidomics**	**Tissue** [85]	Imaging analysis to examine lipidome/lipid profiles, using desorption electrospray ionisation mass spectrometry.	Differentiation of canine cancerous bladder tissue and cutaneous metastasis from noncancerous canine bladder tissue samples. Different lipid distributions between healthy and diseased tissues. DESI-MS imaging could be useful in diagnosing TCC by using a multimarker approach based on the lipid profiles and intensities of tissue samples. Further studies are required with larger populations and additional control groups, i.e., with other lower urinary diseases. Still requires invasive techniques for tissue collection.	N.A.	N.S.	N.S.
	**Urine** [86]	Analysis of lipid profiles using liquid chromatography-mass spectrometry.	Unique lipid profiles were found among dogs with TCC, dogs with UTI, and healthy dogs. Specific statistical analyses allowed their differentiation. Concentrations of the specific lipids could not be determined, and thus the study did not conclude which lipid families were up or downregulated. Foundation for further research on urinary lipids as potential biomarkers for TCC. Non-invasive method.	N.A.	N.S.	N.S.
**Survivin**	**Tissue** [87]	Immunohistochemistry for detection of survivin, an apoptosis-inhibiting protein; RT-PCR analysis for the survivin gene.	Initial phases of investigational development with limited samples. Additional research needed to investigate potential role of nuclear survivin as an early marker for bladder tumours, as well as in the development, progression and as a therapeutic target.	N.A.	N.S.	N.S.
**EGFR**	**Tissue** [88]	IHC and qPCR analysis for EGFR.	EGFR expression could potentially be used as a marker to aid canine TCC diagnosis. It may improve the sensitivity of urine cytological diagnosis when provisional diagnosis is needed.	Not useful for predicting prognosis of TCC.	72%	100%
**HER-2**	**Tissue** [61,89]	IHC for HER-2.	N.A.	Potential maker of malignancy and therapeutic target in canine TCC.	N.S.	N.S.
**VEGFR2,** **PDGFR-β, c-KIT**	**Tissue, cell lines** [90,91,92,93,94]	IHC for expression of VEGFR2, PDGFR-β, c-KIT.	PDGFR-β could play a role in canine TCC tumourigenesis.	PDGFR-β and VEGFR2 might be involved in mediating clinical response of TCC to toceranib.	N.S.	N.S.
**Granzyme B, CD3**	**Tissue** [95]	IHC and PCR assay for CD3 and granzyme B.	N.A.	Granzyme B^+^ tumour-infiltrating cells could be involved in inhibition of tumour progression, and a favourable prognosis. Presence of granzyme B^+^ tumour-infiltrating cells might be an independent prognostic factor.	N.S.	N.S.
**P63, Ki67, β-catenin**	**Tissue** [96,97,98,99,100,101,102]	IHC for p63.	P63 could potentially be used as a clinical marker for diagnosing canine TCC.	P63 could potentially be used as a clinical marker for predicting prognosis in canine TCC.	N.S.	N.S.
**UP III** **CK 7** **CK 20** **COX-2**	**Tissue** [2,44,103,104,105]	IHC for UP III, CK 7 and CK20.	UP III is the most common marker of urothelial differentiation used in dogs. It was considered the marker of choice in canine urothelial neoplasms. Although UP III is not a specific marker for TCC itself (it does not differentiate neoplastic from non-neoplastic lesions), it can be useful e.g., to rule in TCC in a biopsy from a tumour of unknown origin and to identify metastatic carcinomas in the skin. CK 7 was more sensitive than UP III for canine TCC, but CK 7 is expressed in several non-urothelial tumours and also in normal tissues, as is CK 20. CK 7 should be used for tumours negative for UP III but suspected of being TCC. CK 20 alone did not prove to be useful for diagnosis of urothelial tumours. Some urothelial carcinomas might not be positively labelled when using UP III and CK 7 as diagnostic markers. COX-2 has been found to be expressed in canine TCCs but not by normal urothelium of the urinary bladder.	UP III, CK 7, COX-2: Significant associations between specific patterns of expression and tumour classification, depth of neoplastic cell infiltration. COX-2: Intensity of COX-2 expression did not correlate with grading. Nonselective COX and COX-2 specific inhibitors have been used for treating TCC. Still unclear whether it could be useful as a predictive factor for treatment response.	N.S.	N.S.

TCC: transitional cell carcinoma; PC: prostatic carcinoma; N.A.: Not available; N.S.: Not specified; y.o.: years old; vs.: versus.

## Data Availability

No data were generated in this study.

## Supplementary Materials

The following supporting information can be downloaded at: https://www.mdpi.com/article/10.3390/vetsci9030107/s1, Figure S1: Evolution of histological classification and grading schemes for urothelial lesions, Figure S2: TNM clinical staging of canine TCC.
