# Supplementary material for: Molecular Markers in Urinary Bladder Cancer: Applications for Diagnosis, Prognosis and Therapy
Source: Vet Sci. 2022 Feb 28;9(3):107. doi: 10.3390/vetsci9030107 (PMC8950778; doi:10.3390/vetsci9030107)
Supplement: Supplementary file 1 [file vetsci-09-00107-s001.zip › vetsci-1533788-supplementary.pdf]

veterinary sciences

MDPI

Supplementary Materials

Molecular Markers in Urinary Bladder Cancer: Applications for Diagnosis, Prognosis and Therapy

Ana Mafalda Rasteiro, Eva Sá e Lemos, Paula A. Oliveira and Rui M. Gil da Costa

**WHO**	**WHO / ISUP**	**WHO**	**WHO**	**Cheng**	**Animals**
Papilloma	Papilloma	Papilloma	Papilloma	Papilloma	Papilloma
Grade 1	PUNLMP	PUNLMP	PUNLMP	Grade 1 (low)	Low grade (non-invasive)
Grade 2	Low grade	Grade 1	Low grade	Grade 2 (low)	
		Grade 2		Grade 3 (high)	High grade (invasive)
Grade 3	High grade	Grade 3	High grade	Grade 4 (high)	
**1973**	**1998**	**1999**	**2004**	**2012**	**2016**

Figure S1: Evolution of histological classification and grading schemes for urothelial lesions. WHO: World Health Organization; ISUP: International Society of Urological Pathology; PUNLMP: Papillary Urothelial Neoplasm of Low Malignant Potential. Adapted from Cheng et al, 2012 and Meuten and Meuten, 2016 [43,2]. There is no exact correlation between grading systems.

T: Primary Tumour

Tis Carcinoma in situ
T0 No evidence of primary tumour
T1 Superficial papillary tumour
T2 Tumour invading the bladder wall, with induration
T3 Tumour invading neighbouring organs (prostate, uterus, vagina, pelvic canal)

N: Regional Lymph Nodes (RLN - internal and external iliac lymph nodes)

N0 No evidence of RLN involvement
N1 RLN involved
N2 RLN and juxta-regional RLN (lumbar lymph nodes) involved

M: Distant Metastasis

M0 No evidence of distant metastasis
M1 Distant metastasis detected – specify site(s)

Figure S2. TNM clinical staging of canine TCC. Adapted from Owen, 1980 [54].
